# A study of the impacts of motivational regulation and self-regulated second-language writing strategies on college students’ proximal and distal writing enjoyment and anxiety

**DOI:** 10.3389/fpsyg.2022.938346

**Published:** 2022-08-09

**Authors:** Yining Zhang, Lianqi Dong

**Affiliations:** Department of Foreign Languages and Literatures, Tsinghua University, Beijing, China

**Keywords:** motivational regulation, self-regulated writing strategies, enjoyment, anxiety, self-regulated learning

## Abstract

Motivational regulation is crucial to explaining autonomous self-regulated learning, yet has received relatively little empirical attention. This study therefore examined how 230 college students’ motivational-regulation strategies affected their proximal and distal second-language writing-achievement emotions (i.e., enjoyment and anxiety), and sought evidence of interactive effects of such strategies and self-regulated learning strategies on each of these two types of emotions. All the studied types of motivational-regulation strategy were found to directly predict both proximal and distal writing enjoyment, under a “the more the happier” principle, but only a performance-oriented motivational regulation strategy predicted proximal or distal writing anxiety. A social-behavior learning strategy was found to counteract the high proximal anxiety caused by heavy use of the performance self-talk motivational regulation strategy; and motivational-regulation predictors also emerged as stable predictors of both proximal and distal writing well-being. These findings are expected to be both theoretically valuable to the study of motivational regulation under the self-regulated learning framework, and of practical value to educators, learners, and curriculum designers.

## Introduction

Self-regulated learning (SRL) is an autonomous individual-level learning process that entails a combination of motivation, cognitive skills, and metacognitive skills (Zimmerman, 1986, 2011), along with active and purposeful management of one’s own motivations, i.e., motivational regulation (Wolters, 1998). Although motivational regulation is widely accepted theoretically, it remains a relatively under-studied component of SRL (Schwinger et al., 2009; Grunschel et al., 2016). Moreover, studies of it conducted among students have primarily examined its relationship to learning achievement, while ignoring its emotional impacts, despite findings that students’ emotions during the learning process affect their subsequent learning experience (Pekrun, 2006; Pekrun and Perry, 2014). Although positive emotions generally tend to be correlated with higher achievement, and negative emotions with lower achievement, the relations among these four constructs are more complex than they at first appear (see Pekrun and Linnenbrink-Garcia, 2012 for a review). Therefore, it should not simply be assumed that what applies to learning performance will also apply to achievement emotions. Indeed, a more nuanced understanding of how motivational regulation and achievement emotions affect one another will help to refine the SRL framework.

In addition, although self-regulated second language (L2) writing strategies have been found to function as a mediator between motivational regulation strategies and writing performance (Teng and Zhang, 2018), it is not clear whether this additional role strengthens the relationship between motivational-regulation strategies and writing emotions. Thus, the secondary aim of this study is to test for any moderating role(s) of self-regulated L2 writing strategies in the prediction of emotions. And thirdly, this study will examine whether the identified predictors of L2 writing well-being can stably predict both proximal and distal well-being.

## Literature review

### Self-regulated learning strategies in writing

The 21st century is an era of autonomous and life-long learning, where learners advance with increasing technologies and expanded opportunities and are supposed to take more charge of their learning process. This has brought to the notion of self-regulated learning (SRL), or self-regulation pioneered by the work of Zimmerman (1986) in educational psychology. As defined, self-regulation refers to the processes whereby learners personally activate and sustain cognitions, affect, and behaviors that are systematically oriented toward the arraignment of personal goals (Zimmerman and Schunk, 2011). Accumulating research has evidenced that SRL strategies enable individuals to manage their strategic learning, achieve better and have other positive developmental outcomes (e.g., Zheng et al., 2018).

Self-regulation is domain- and context-dependent. One area that has drawn considerable scholarly attention in recent years is SRL strategies in writing. Reasons for empowering learners to self-regulate their writing process are well-grounded. As one of the most challenging tasks in learning, writing reflects not only learners’ overall linguistic competence and knowledge repertoire (Anastasiou and Michail, 2013) but also is a hierarchically structured process subject to the dynamic interactions of a wide range of environmental and individual factors (Flower and Hayes, 1981). It is found that SRL writing strategies lead to enhanced writing engagement, products and skills (Hayes, 2000).

Whereas SRL writing strategies may be a familiar inquiry in L1 (first language), it is a relatively new concept in the context of L2. Drawing on the work from both educational psychology and applied linguistics, Teng and Zhang (2016b) first conceptualized SRL writing strategies as deliberate, goal-directed attempts to make writing enjoyable, less challenging, and more effective, and designed the SRL writing strategies questionnaire that measure three types of SRL writing strategies, including cognitive, metacognitive, and social behavior strategies (Teng and Zhang, 2018). Following Teng and Zhang (2016b,2018), researchers attempted to identify SRL writing strategies’ antecedents, moderators, and outcomes. Existing studies on SRL writing strategies in L2 settings have revealed that writing corrective feedback orientations and mindsets significantly predicted the use of SRL writing strategies (Xu, 2022); that SRL writing strategy use differed across gender, language proficiency, and grade level (Teng and Huang, 2019; Bai et al., 2020); and that SRL writing strategies contribute significantly to students’ writing self-efficacy and proficiency (Teng and Huang, 2019; Sun and Wang, 2020). Although the role of SRL writing strategies has been sufficiently recognized, our interpretation of it is nevertheless limited in scope. To date, an overwhelming proportion of studies that followed this inquiry have concerned how the use of SRL writing strategies predicted writing performance as measured by cross-sectional data of mere writing scores, with scant attention paid to the nature of writing which is also an affective and social process under the influence of the interacting factors (Harris et al., 2011).

### Motivational-regulation strategies

According to Wolters (1998), purposeful management of one’s own motivations, namely, motivational regulation, is a key component in SRL. Wolters (2003) and Schwinger et al. (2009) define motivational regulation as deliberate actions taken to initiate, adjust, increase, or maintain one’s own willingness to start, persist in, and complete a learning task. Motivational regulation helps mobilize cognitive, metacognitive, and social strategies, facilitate learning and improve academic achievement (Schunk and Zimmerman, 2008; Teng and Zhang, 2016b). Thus, it is believed to be an integral part of SRL, given that it addresses learners’ active management of their learning experience in a variety of ways (Zimmerman, 2002; Sansone and Thoman, 2006), which are presumably not restricted to cognitive and metacognitive strategies only (Wolters, 1998). Inspired by the need to understand the complete processes where students regulate their motivational states in academic goal pursuit, Miele and Scholer (2018) proposed a metamotivational model of motivation regulation by building on the work of previous theorists. As conceptualized, successful motivation regulation during task completion requires students to fully utilize metamotivational knowledge (i.e., accurate beliefs about how motivation functions) to initiate and maintain metamotivational monitoring and control processes which entail many reciprocal subprocesses that are cognitive, metacognitive, motivational and emotional (Miele and Scholer, 2018; Miele et al., 2020).

Prior SRL studies, however, have seldom treated motivational regulation as a distinct construct but rather as integral to students’ processes of controlling and managing their learning (Wolters and Rosenthal, 2000). Thus, various strategies further described below, including self-consequating (Zimmerman and Martinez-Pons, 1986), environmental structuring (Zimmerman and Martinez-Pons, 1986; Pintrich, 1999), and self-handicapping (Garcia and Pintrich, 1994), have been presented as reflections of students’ efforts to manage their motivation. Efforts to explicitly measure and study learners’ acts of motivational regulation began with Wolter’s (1998) development of a robust questionnaire for capturing the relevant strategies, which is grouped into five main areas. These were self-consequating (self-provided extrinsic stimuli); environmental control (the alleviation of distractions); performance self-talk (the tendency to focus motivation on external outcomes); mastery self-talk (the tendency to focus motivation on knowledge mastery); and interest enhancement (regulations to make learning tasks more enjoyable). The validity of the questionnaire was confirmed in a series of publications by Wolter and colleagues.

Explicit classifications of motivational regulation strategies have fueled the development of motivational regulation research in general education settings and other domain/task-specific fields. However, the debate on the effects of motivational regulation strategies seems raging for a long time, mostly expressed on whether and which motivational regulation strategies predict academic outcomes. Some researchers argued that motivational regulation strategies directly predict learning achievement (e.g., Wolters, 1999; Seker, 2016). For example, Seker (2016) found different motivational regulation orientations could positively or negatively impact Turkey students’ academic performance. By contrast, other researchers believed the effects of motivational regulation strategies on learning achievement were more likely to be indirect (Schwinger et al., 2009; Schwinger and Stiensmeier-Pelster, 2012; Grunschel et al., 2016). While such differences may be pinned down to contextual variations, it is important to note that the working mechanism of motivational regulation strategies can be rather complex which may be influenced by other individual factors.

Regarding the impact of specific motivational-regulation strategies on learning, most research findings favor the use of mastery self-talk strategies over performance self-talk, in keeping with goal achievement theory (Hulleman et al., 2010; Schwinger and Stiensmeier-Pelster, 2012). Despite this, others argued that performance self-talk could also be an essential contributor to the learning process and successes since it is more concerned with learning outcomes (see in Teng and Zhang, 2016a) so that in specific learning context performance self-talk may serve as a strong motivational impetus. Similarly, in their recent discussion of metamotivational knowledge in motivation regulation, Miele et al. (2020) maintained that both promotion and prevention motivations can be conducive to task completion, with the former more helpful for associative, divergent, and flexible thinking while the latter more beneficial to concrete, convergent and careful thinking.

When viewing motivational regulation strategies in L2 writing, Teng et al. (2020) argued that they are crucial to L2 writing, because it is not likely that L2 learners could secure long-term success only with cognitive and metacognitive strategies given writing as a social cognitive process. For this reason, L2 writing needs to be situated within a dynamic motivational state (Troia et al., 2013). Teng and Zhang (2016a) validated a measurement instrument for motivational regulation in L2 writing that included four types of strategies: motivational self-talk, interest enhancement, emotional control and environment structuring. Empirical findings have evidenced that high writing proficiency students tended to use more motivational self-talk, interest enhancement, and emotional control than their low writing-proficiency peers (Teng et al., 2020) and that motivational regulation directly or indirectly predicted writing performance (Teng and Zhang, 2018). Despite increasing recognition of the role of motivational regulation in L2 writing, little is known about its effects on other variables that emerge in the dynamic process of writing except for academic outcomes, such as affective and social factors which are equally essential to the writing process regulation.

### Interactions between self-regulated learning strategies and motivational regulation strategies

The crucial role of both self-regulated learning and motivational regulation in the learning process and the highlighted motivational regulation in most SRL frameworks reasonably warrant the possibility that aside from being integral to self-regulation, motivational regulation strategies may interact with SRL strategies in practice. As theoretically claimed by some researchers (e.g., Pintrich, 2004; Schwinger et al., 2009), motivational regulation strategies may work simultaneously with other SRL strategies to maintain and enhance the learning process and goal achievement. Empirically, research has indicated that the use of motivational regulation strategies could serve as an important antecedent of and account for the variance of individuals’ SRL strategy use (Wolters, 1999; Teng and Zhang, 2018). Therefore, it seems unarguable that SRL strategies and motivational regulation strategies contribute distinctly to the learning process and that their interaction merits further investigation.

The only study to date to have explicitly investigated the interaction between motivational regulation and SRL strategies, by Nguyen and Deci (2016), found an interactive effect of controlled motivational regulation and setting high standards on test anxiety. More specifically, setting high standards was correlated with high test anxiety when a student experienced high extrinsic motivational regulation. To our best knowledge, no research to date has examined the interactive effects of SRL strategies and motivational regulation strategies in the context of L2 learning. Given the important role of self-regulated strategies in the learning process, more studies of the possible moderating effects of motivational regulation strategies are warranted.

### Achievement emotions as important indicators of learning and wellbeing

Learning is a multi-faceted activity. The exclusive pursuit of high achievement performance can be detrimental for the learning process to be fundamentally sustainable. Thus, it is paramount for educational practitioners to value learner development and wellbeing in which emotions play a backbone role (William and Hoffman, 2020). Achievement emotions are affective arousal tied directly to achievement activities (Pekrun and Perry, 2014). According to the control-value theory (Pekrun, 2006), individuals’ experience of achievement emotions is the product of to what extent they feel in control of and subjectively value the task or activity. Interestingly, it seems evident that the two antecedental appraisals of achievement emotions correspond to some major components of established motivational models, such as expectancy-value theories of motivation. Hence, it is logical to speculate that the motivational regulation, apart from predicting the well-documented learning proficiency, may possibly predict achievement emotions, which, if adequately addressed, is likely to yield revealing pedagogical implications.

Yet, despite a range of literature showing that the use of particular motivational-regulation strategies can predict learning performance, very few studies have explored the relationship between motivational regulation and achievement emotions. Among the limited studies, Fritea and Fritea (2013) explored the relationship between motivational-regulation strategies and boredom, and found that the latter construct was correlated negatively with both regulations of value and regulation of performance goals. Park and Yun (2018) found that the adoption of a mastery self-talk strategy was the only significant predictor of online students’ emotional engagement. However, the construct of emotional engagement in that study captured only the excitement and enjoyment of learning, and negative emotions such as anxiety were not investigated.

Indeed, both enjoyment and anxiety are important emotional indicators in L2 learning (Dewaele, 2021a). However, little is known about whether, how, or how much individual differences in motivational-regulation levels are correlated with differences in enjoyment and anxiety. Based on findings to date, it would appear that motivational regulation strategies which are saliently marked by achievement goal orientations are closely tied to both enjoyment and anxiety (King et al., 2012); that having mastery goals is correlated with positive emotions such as enjoyment (e.g., Linnenbrink-Garcia and Barger, 2014); and that having performance goals may or may not be correlated with negative emotions such as anxiety (Huang, 2011; Linnenbrink-Garcia and Barger, 2014). Due to the nature of motivational regulation, our study adopts these prior studies’ conceptualizations of the relationship between achievement goals and discrete emotions. We expect to see a similar pattern emerge when testing motivational-regulation strategies. Moreover, given the interactions between motivational regulation strategies and SRL strategies as reviewed earlier, we are also interested in finding out whether such interactions exist in predicting learner emotions L2 writing.

The present study’s main aim is to examine if, and to what degree, university EFL students’ motivational-regulation strategies, as well as potential interactions between such strategies and their self-regulated writing strategies, predict L2 writing enjoyment and L2 writing anxiety. Its secondary aim is to obtain evidence about the patterns of predictive power of motivational-regulation strategies on both proximal and distal achievement emotions. Specifically, it will be guided by the following research questions:

(1) How do the various motivational-regulation strategies predict proximal and distal L2 writing emotion?
(2) Do self-regulated writing strategies moderate the relationship between motivational regulation strategies and the studied proximal and distal L2 writing emotions?

## Materials and methods

### Participants and context

This study was conducted in four EFL writing courses at a prestigious university in China. A total of 230 students participated, among whom 91 were female and 139, male. There were 56 undergraduates and 174 graduate students, and the average age was 23.43 (*SD* = 0.28). Arts majors made up 36% of the sample, and the rest were majoring in science. One-third (33.5%) self-rated English their writing proficiency at the beginning of the semester as poor or very poor; 53.4% rated it as average; 12.6% as good; and just one individual as very good.

### Procedure

Writing-related course data were collected at two points during the semester. The first of these data-collection rounds (T1) was in week 8, the middle of the semester, and the second (T2) in week 16, its final week. On each occasion, a gatekeeper passed hard copies of our questionnaire to students and collected them once they had been completed.

### Measures

The same questionnaire was administered in each data-collection round, and was composed of three parts, (1) a consent form, (2) 71 items on the respondents’ self-regulated English writing, all answered using a 5-point Likert scale ranging from 1 = “very unlikely” to 5 = “very likely”; and their background information. Supplementary Appendix I provides a copy of the questionnaire.

#### Motivational-regulation strategies

Our study’s focal motivational-regulation strategies were the same as those studied by Teng and Zhang (2018), from whose survey and informed by Teng et al. (2020) who found motivational self-talk, interest enhancement, and emotional control to be the most relevant and proficiency distinguishing dimensions in L2 writing motivation regulation, we also adapted our 14 survey items about such strategies. These items collectively covered four dimensions: mastery self-talk (three items, α = 0.73), performance self-talk (four items, α = 0.81), interest enhancement (four items, α = 0.90), and emotional control (three items, α = 0.66).

#### Second-language self-regulated writing strategies

Our 21 items for measuring L2 students’ self-regulated writing strategies were also adapted from Teng and Zhang’s (2018) instrument. These items covered five dimensions: text processing (five items, α = 0.79), idea planning (three items, α = 0.64), goal-oriented monitoring (six items, α = 0.85), peer learning (three items, α = 0.76), and feedback-handling (four items, α = 0.76). Of these five dimensions, text processing is a cognitive strategy; idea planning and goal-oriented monitoring, metacognitive strategies; and peer learning and feedback-handling, social-behavior strategies.

#### Writing-achievement emotions

Our seven items for measuring achievement emotions were adapted from the Academic Emotions Questionnaire (AEQ) developed by Pekrun et al. (2005). As well as translating them into Chinese, we reworded some of these items to make them more appropriate for measuring emotions about writing. The original AEQ covers achievement emotions in three domains (i.e., class, learning, and test), but we only included learning-related items, as most closely reflecting our study’s primary goal. Anxiety was measured by three items (α = 0.70 at T1, α = 0.72 at T2), and enjoyment was measured by four (α = 0.73 at T1, α = 0.77 at T2).

### Data analysis

Before statistical analyses, we ran power analysis in G*Power software (Version 3.1.9.6) to test whether our sample size (*n* = 230) was large enough to allow subsequent statistical inference by keeping the rigorous threshold of effect size, significant level and power (Cohen’s *d* = 0.5, α = 0.05, 1 − β = 0.95) (Faul et al., 2009), respectively. The result showed that to meet these standards, a sample had to contain at least 176 participants, meaning that our sample size was suitable for statistical analyses. Prior to answering our research questions, we explored the factor structure of the two independent variables, i.e., 14 motivational-regulation strategies and 21 self-regulated writing strategies. This enabled us to reduce multicollinearity when conducting multiple regressions. Principal component analysis (PCA) with varimax rotation was used to extract the major components of the measures of each strategy. The results of PCA are presented in the “Results” section, following the discussion of the descriptive statistics.

Once we had established the major components of the motivational-regulation and self-regulated writing strategies, we used hierarchical multiple regression analysis to answer our research questions. Interaction terms, created to capture the moderating process of interest, were entered into the regression models. Following the finding of a significant interaction, we conducted additional simple slope analysis (Cohen et al., 2013).

## Results

### Descriptive statistics

Table 1 shows the means, standard deviations, and correlations of the composite scores of motivational-regulation strategies, self-regulated writing strategies, writing anxiety at T1 and T2, and writing enjoyment at T1 and T2. All six variables showed moderately high scores. We then observed the first-order correlations among them. In keeping with previous research, students who used motivational-regulation strategies more often also tended to report more use of self-regulated writing strategies, as well as higher enjoyment at both time points. There was no association between motivational-regulation strategies and anxiety at either time point, but enjoyment and anxiety were negatively correlated with each other. Additionally, self-regulated writing strategies correlated negatively with anxiety and positively with enjoyment. The correlations between enjoyment at two time points, and between anxiety at two time points were positive and significant, indicating the stability of the emotion over time.

**TABLE 1:** Descriptive statistics and correlations before principal component analysis (*N* = 230).

Variable	M	SD	Skewness	Kurtosis	1	2	3	4	5	6
(1) T1 MR	3.72	0.53	–0.10	3.52	1					
(2) T1 SRL	3.83	0.43	0.18	0.21	0.79***	1				
(3) T1 ANX	3.23	0.76	–0.43	3.17	–0.09	−0.18**	1			
(4) T1 ENJ	3.55	0.64	–0.08	3.44	0.77***	0.65***	−0.16*	1		
(5) T2 ANX	3.24	0.78	–0.15	2.72	–0.02	–0.09	0.58***	–0.09	1	
(6) T2 ENJ	3.68	0.64	–0.17	3.31	0.70***	0.64***	−0.21**	0.75***	–0.09	1

MR, motivational-regulation strategies; SRL, self-regulated writing strategies; ANX, writing anxiety; ENJ, writing enjoyment.

*p < 0.05; **p < 0.01; ***p < 0.001.

### Principal component analysis

Two sets of PCA analyses with varimax rotation were conducted, one to find factor solutions for motivational-regulation strategies, and the other to find them for self-regulated writing strategies. The first set of PCA results yielded a two-component solution with both factors’ eigenvalues larger than 1 (i.e., 6.12 and 1.35), which explained 53.38% of the total variance. The factor loadings for motivational-regulation strategies are displayed in Table 2. We used a loading criterion of 0.40, as recommended by Floyd and Widaman (1995), to decide which items should be included under each factor. The first component contained all items designed for measuring the students’ interest enhancement, mastery self-talk, and emotional control, with factor loadings ranging from 0.49 to 0.78. We labeled this component as *Intrinsic and mastery motivational-regulation strategies*. The second component included the four items for measuring Performance self-talk, with factor loadings ranging from 0.63 to 0.87, and was labeled as *Performance self-talk*.

**TABLE 2:** Principal component analysis factor loadings for motivational regulation.

Item	Component 1	Component 2
Interest enhancement 1	**0.49**	0.30
Interest enhancement 2	**0.76**	0.10
Interest enhancement 3	**0.78**	0.15
Interest enhancement 4	**0.71**	0.16
Mastery self-talk 1	**0.53**	0.37
Mastery self-talk 2	**0.67**	0.39
Mastery self-talk 3	**0.61**	0.35
Emotional control 1	**0.55**	0.24
Emotional control 2	**0.68**	0.32
Emotional control 3	**0.56**	0.33
Performance self-talk 1	0.32	**0.63**
Performance self-talk 2	0.36	**0.68**
Performance self-talk 3	0.10	**0.87**
Performance self-talk 4	0.15	**0.83**

|Loadings| > 0.40 are displayed in bold. Each measurement item can be seen in the Supplementary Appendix I.

Similarly, we conducted PCA on all the items measuring self-regulated writing strategies (see Table 3). The results suggested a four-component solution with all eigenvalues greater than 1 (i.e., 6.98, 2.44, 1.80, and 1.13), which explained 58.81% of the variance. The first component contained all six variables from goal-oriented monitoring, plus Idea planning 3 and Peer learning 1. However, both Idea planning 3 and Peer learning 1 loaded onto more than one component with factor loadings larger than 0.4, and had higher factor loadings on the fourth component and third component, respectively. Therefore, Idea planning 3 and Peer learning 1 were excluded from the first component. We labeled component 1 as *Goal-oriented monitoring strategy*. Following Teng and Zhang (2018), we named the second component *Cognitive strategy*, as it mostly involved students’ abilities to process cognitive information in writing: i.e., consisted of all five items for measuring text processing, plus Feedback-handling 2 and 4. The third component included all items designed to measure Peer learning and Feedback-handling. Again, as this component echoed Teng and Zhang’s (2018) findings, we used their label for it: *Social-behavior strategy*. The last component contained all items from Idea planning strategy, and we therefore decided to label it with that term.

**TABLE 3:** Principal component analysis factor loadings for self-regulated writing strategies.

Item	Component 1	Component 2	Component 3	Component 4
Text processing 1	0.04	**0.68**	0.07	0.22
Text processing 2	0.08	**0.63**	0.02	0.08
Text processing 3	0.17	**0.66**	0.19	0.11
Text processing 4	0.16	**0.71**	0.00	0.19
Text processing 5	0.22	**0.73**	0.11	0.04
Idea planning 1	0.35	0.24	0.01	**0.65**
Idea planning 2	0.08	0.19	0.20	**0.74**
Idea planning 3	**0.49**	0.38	0.06	**0.40**
Goal-oriented monitoring 1	**0.74**	0.07	0.04	0.15
Goal-oriented monitoring 2	**0.69**	0.07	0.19	0.33
Goal-oriented monitoring 3	**0.69**	0.24	0.16	0.03
Goal-oriented monitoring 4	**0.67**	0.29	0.17	0.12
Goal-oriented monitoring 5	**0.60**	0.38	0.19	−0.21
Goal-oriented monitoring 6	**0.75**	0.00	0.25	0.08
Peer learning 1	**0.46**	−0.14	**0.57**	0.23
Peer learning 2	0.43	−0.14	**0.56**	0.29
Peer learning 3	0.32	0.08	**0.74**	−0.08
Feedback-handling 1	−0.05	0.25	**0.74**	0.29
Feedback-handling 2	−0.14	**0.49**	**0.56**	0.13
Feedback-handling 3	0.27	0.11	**0.77**	−0.07
Feedback-handling 4	0.14	**0.58**	**0.44**	0.13

|Loadings| > 0.40 are displayed in bold. Each measurement item can be seen in the Supplementary Appendix I.

### The main effects of motivational-regulation strategies on writing-achievement emotions

A series of stepwise multiple regression analyses were conducted to find main effects of motivational regulation and self-regulated writing strategies on writing emotion. We computed eight variables that captured the interaction between, on the one hand, two motivational-regulation variables (i.e., *intrinsic and mastery motivational-regulation strategies* and performance self-talk), and on the other, four self-regulated writing strategies (i.e., goal-monitoring, cognitive strategies, social-behavior, and idea planning). The complete regression model’s set of independent variables thus consists of two motivational-regulation variables, four self-regulated writing strategies, and eight interaction variables, as well as four control variables: i.e., gender, major, grade, and self-rated writing proficiency. The four dependent variables were T1 enjoyment, T2 enjoyment, T1 anxiety and T2 anxiety, respectively. All independent variables were centered to avoid non-essential multicollinearity (Cohen et al., 2013).

In the case of T1 enjoyment, as shown in Table 4, Model 1 – which included motivational-regulation strategies and self-regulated writing strategies but not the interaction between the two – was found to be significant, with *R*^2^ = 0.64, *F*(10, 197) = 34.54, *p* < 0.001. *Intrinsic and mastery motivational-regulation strategies* (*b* = 0.37, *p* < 0.001) and *Performance self-talk* (*b* = 0.20, *p* < 0.001) both predicted T1 enjoyment positively, and these effects remained significant after the eight interaction variables were added, in Model 2 (*Intrinsic and mastery motivational-regulation strategies b* = 0.37, *p* < 0.001, and *Performance self-talk (b* = 0.21, *p* < 0.001). Model 2 yielded *F*(18, 189) = 19.53, *p* < 0.001 with *R*^2^ = 0.65.

**TABLE 4:** The main and interaction effects of motivational-regulation strategies and self-regulated writing strategies on writing enjoyment at two time points.

	T1 Enjoyment				T2 Enjoyment			
		
	Model 1		Model 2		Model 1		Model 2	
				
	*b*	*SE.b*	*b*	*SE.b*	*b*	*SE.b*	*b*	*SE.b*
MS	0.37***	0.05	0.37***	0.05	0.28***	0.05	0.28***	0.06
PS	0.20***	0.03	0.21***	0.04	0.16***	0.04	0.19***	0.04
GM	0.06	0.04	0.06	0.04	0.09	0.05	0.07	0.05
COG	–0.01	0.03	–0.01	0.04	0.08	0.04	0.06	0.04
SB	–0.00	0.03	0.01	0.04	0.08	0.04	0.09*	0.04
IP	0.05	0.03	0.03	0.03	0.07*	0.04	0.05	0.04
MS × GM			0.01	0.03			–0.01	0.03
PS × GM			0.00	0.02			0.02	0.03
MS × COG			0.02	0.03			0.01	0.03
PS × COG			–0.04	0.03			–0.06	0.03
MS × SB			0.03	0.02			0.05	0.03
PS × SB			0.01	0.03			0.00	0.03
MS × IP			–0.04	0.02			–0.04	0.03
PS × IP			0.01	0.02			0.04	0.03
*R* ^2^	0.64		0.65		0.54		0.56	

COG, cognitive strategy; GM, goal-oriented monitoring strategy; IP, idea planning strategy; MS, intrinsic and mastery motivational-regulation; PS, performance self-talk; SB, social-behavior strategy. Control variables: gender, age, major, and self-rated writing proficiency.

*p < 0.05; ***p < 0.001.

In the case of T2 enjoyment, Model 1 suggested that *Intrinsic and mastery motivational-regulation* (*b* = 0.28, *p* < 0.001) and *Performance self-talk* (*b* = 0.16, *p* < 0.001) again were positive and significant predictors (see Table 4). And, after adding the interaction variables in Model 2, *Intrinsic and mastery motivational-regulation* (*b* = 0.28, *p* < 0.001) and *Performance self-talk* (*b* = 0.19, *p* < 0.001) remained significant predictors of this dependent variable.

We then tested the main effect of motivational-regulation strategies on T1 anxiety. As shown in Table 5, both Model 1 and Model 2 suggested that T1 anxiety was only linked to *Performance self-talk* (*b* = 0.18, *p* < 0.01 in Model 1, and *b* = 0.15, *p* < 0.05 in Model 2), and not to *Intrinsic and mastery motivational-regulation*. The same pattern also applied in the prediction of T2 anxiety, with *b* = 0.22, *p* < 0.01 in Model 1, and *b* = 0.17, *p* < 0.05 in Model 2.

**TABLE 5:** The main and interaction effects of motivational-regulation strategies and self-regulated writing strategies on writing anxiety at two time points.

	T1 anxiety				T2 anxiety			
		
	Model 1		Model 2		Model 1		Model 2	
				
	*b*	*SE.b*	*b*	*SE.b*	*b*	*SE.b*	*b*	*SE.b*
MS	0.02	0.08	0.01	0.16	−0.01	0.09	−0.00	0.09
PS	0.18**	0.06	0.15*	0.10	0.22**	0.07	0.17*	0.07
GM	−0.07	0.07	−0.03	0.07	0.00	0.08	0.06	0.08
COG	−0.12*	0.06	−0.13*	0.06	−0.19**	0.07	−0.19**	0.07
SB	0.03	0.06	0.03	0.06	−0.03	0.06	−0.02	0.07
IP	−0.12*	0.05	−0.08	0.05	0.08	0.06	0.12*	0.06
MS × GM			0.00	0.11			0.07	0.05
PS × GM			−0.06	0.10			−0.08	0.04
MS × COG			−0.02	0.11			−0.04	0.05
PS × COG			−0.02	0.10			−0.03	0.05
MS × SB			−0.07	0.08			−0.06	0.04
PS × SB			−0.10*	0.08			−0.07	0.05
MS × IP			0.04	0.10			0.03	0.04
PS × IP			−0.06	0.08			−0.08	0.04
*R* ^2^	0.26		0.31		0.19		0.25	

COG, cognitive strategy; GM, goal-oriented monitoring strategy; IP, idea planning strategy; MS, intrinsic and mastery motivational-regulation; PS, performance self-talk; SB, social-behavior strategy. Control variables: gender, age, major, and self-rated writing proficiency.

*p < 0.05; **p < 0.01.

### The moderating effect of self-regulated writing strategy

To test whether self-regulated writing strategies moderated the relationship between motivational-regulation strategies and writing emotions, the interaction effects were added as Model 2 when predicting enjoyment and anxiety, respectively. As shown in Table 5, there was a marginally significant interaction effect of *Performance self-talk* and *Social-behavior strategy* on the prediction of writing anxiety at T1 (*b* = −0.10, *p* < 0.05).

We then conducted simple slope analysis (Cohen et al., 2013) to test the significance of the slopes of *Performance self-talk* in relation to T1 anxiety at one standard deviation below and above the mean of *Social-behavior strategy*. The results suggested that, when a given student’s *Social-behavior strategy* and *Performance self-talk* were both high, this was associated with a slight increase in T1 anxiety (*t* = 0.65, *p* > 0.05). However, that relation was not significant. On the other hand, when a student’s *Social-behavior strategy* was low, the link between that person’s *Performance self-talk* and his/her T1 anxiety was positive and significant (*t* = 2.37, *p* < 0.05).

**FIGURE 1:**
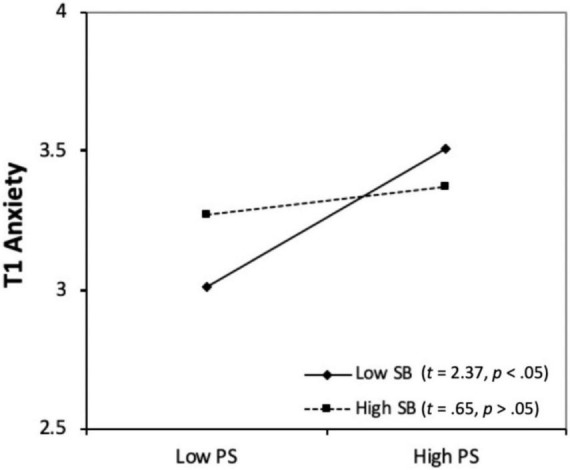
Social-behavior strategy as a moderator between performance self-talk and T1 Anxiety. PS, performance self-talk; SB, social-behavior.

The interaction relationships are further illustrated in Figure 1. Although one of the two slopes is not significant, the overall pattern suggests that the less *Social-behavior strategy* students used, the stronger was the positive relationship between their *Performance self-talk* and their T1 anxiety. Conversely, if two students both reported low levels of *Performance self-talk*, the one with the higher level of *Social-behavior strategy* tended to show a higher level of T1 anxiety. However, this situation was reversed when both students’ use of *Performance self-talk* was rated as high: with the one having a lower level of *Social-behavior strategy* also exhibiting a higher level of T1 anxiety.

### Change over time

We then examined patterns of stability in the prediction of enjoyment and anxiety. Both *Intrinsic and mastery motivational-regulation strategies* and *Performance self-talk* showed decreases over time in their power to predict enjoyment. However, these decreases in effect size were slight: i.e., from *b* = 0.37 at T1 to *b* = 0.28 at T2 for the former, and from *b* = 0.21 at T1 to *b* = 0.19 at T2 for the latter (see Table 4). In addition, *Social-behavior strategy* was not associated with enjoyment at T1, but nevertheless emerged as a significant predictor of distal enjoyment (*b* = 0.09, *p* < 0.05).

The pattern of stability in the prediction of anxiety is somewhat different. Of the two motivational-regulation variables, only *Performance self-talk* predicted anxiety at both time points, and showed a slight increase in its predictive power: from *b* = 0.15 to *b* = 0.17. *Cognitive strategy* also appeared as a stable predictor over time, with *b* = −0.13 at T1, and *b* = −0.19 at T2. *Idea planning strategy* was a significant predictor only of distal anxiety, with *b* = 0.12 (see Table 5). Lastly, the interaction between *Performance self-talk* and *Social-behavior strategy* in the prediction of anxiety was significant at T1 (*b* = −0.10, *p* < 0.05), but not at T2 (*b* = −0.07, *p* > 0.05).

## Discussion

The SRL framework depicts the motivational, metacognitive, cognitive, and behavioral mechanisms of individuals’ active learning (Bandura, 1986; Zimmerman, 2002). Within such a framework, motivational regulation is helpful in explaining a person’s autonomous role in initiating, adjusting, increasing, or maintaining his or her own motivation (Wolters, 1998), and yet, it has been the subject of relatively few empirical investigations. Our study has made important contributions to this largely overlooked area. Its theoretical contributions, practical implications, and limitations and future directions are discussed below.

The findings from our study support, challenge, and extend various results reported by previous researchers regarding the role of motivational regulation in the SRL framework. Like Schwinger et al. (2007), we identified two distinct groups within the set of measured motivational-regulation strategies, one containing only performance self-talk strategies, and the other, all other such strategies. Our two-factor solution derived from PCA analysis is consistent with previous studies’ dichotomous categorizations of students’ goal-oriented motivational regulation (Wolters, 1998; Wolters and Rosenthal, 2000), which differentiated mastery self-talk from performance self-talk; and with Schwinger et al.’s (2007) intrinsic vs. extrinsic classification. Our finding is also in line with the distinction educational psychologists have drawn between, on the one hand, more mastery- and intrinsic-oriented learning goals, and on the other, more performance- and extrinsic-oriented ones (Deci and Ryan, 1985; Elliot, 1999).

Although little research has examined motivational regulation as a predictor of emotion, it is important to note that our results differ somewhat from Park and Yun’s (2018) finding that mastery self-talk was the only significant predictor of emotional engagement. That is, we found that all types of motivational-regulation strategies were significantly linked to writing enjoyment, which seems to confirm Schwinger et al. s’ (2012) comment that motivational regulation follows a “the more, the merrier principle” (p. 277). Our result also provides empirical support for Zimmerman and Schunk’s (2008) hypothesis that students’ motivational regulation may have positive impacts on their affective outcomes; and extends our understanding of such a process, in that it is not any specific type of motivational regulation that matters in predicting positive affective outcomes, but the overall amount. Our finding that learners’ use of performance self-talk is not necessarily detrimental, meanwhile, is in accordance with the findings of previous educational-psychology research on the positive relationship between performance goals and positive emotions (Barron and Harackiewicz, 2001; Pekrun, 2006; Bodmann, 2009). Also, it should be noted that the specific context of our study – an elite Chinese university – was a competitive learning environment in which the participants inevitably valued their own high performance. These conditions could have had an effect on the role of performance self-talk, i.e., rendered it conducive to eliciting a sense of enjoyment, in a way that it might not in less-competitive or non-competitive settings.

Our results regarding the prediction of anxiety showed that, among all the studied motivational-regulation strategies, only performance self-talk functioned as a significant predictor of this emotion; and specifically, that a high level of performance self-talk was associated with high writing anxiety. This finding is not surprising, given that those people with strong desires to outperform others can reasonably be expected to feel anxious when thinking about their external learning goals. As such, our findings provide additional evidence supporting the previously observed relationship between maladaptive learning outcomes and performance self-talk (Wolters, 1998; Wolters and Rosenthal, 2000; Fritea and Fritea, 2013; Teng and Zhang, 2016a). That is, focusing on external rewards, punishments, or appraisals may be detrimental to an individual’s learning outcomes (Zimmerman and Schunk, 2008), largely because his or her basic psychological need for autonomy is thwarted by a performance-oriented style of motivation dominated by what “must” be done (Nguyen and Deci, 2016, p. 249). As Schwinger and Stiensmeier-Pelster (2012) explained, a student is highly likely to become stressed if an activity is regulated only by an extrinsic motivation, because “there is no positive phenomenological experience while completing the task” (p. 37), and as a result, will inevitably either terminate the learning task or exhibit lower performance than others with intrinsic motivations. In short, the findings of our study imply that educators and educational institutions should encourage the use of mastery-, emotional-, and interest-oriented motivational-regulation strategies over performance-oriented ones, not least as a means of helping their students maintain a sense of well-being.

We also found that social-behavior strategy moderated the relation between motivational regulation and proximal learning anxiety. Remarkably, greater use of social-behavior strategy (i.e., help-seeking and feedback-handling) weakened the positive relationship between performance self-talk and anxiety, while a lesser use of social-behavior strategy strengthened that relationship. As well as tending to confirm the triadic interrelationship of behavioral, environmental, and cognitive factors proposed by the social-cognitive perspective (Bandura, 1986), these findings reflect the learner’s role as an active agent who seeks help and handles feedback during his or her learning process (Schunk and Zimmerman, 1998; Zimmerman, 2002). They also provide empirical support for Newman’s (2012) theoretical conceptualization of action-to-need patterns: i.e., that a student who exhibits little help-seeking behavior is more likely to have performance-approach goals and to be anxious.

In addition to providing empirical support to the existing body of theory, our social-behavior findings serve to explain why Teng and Zhang (2018) found cognitive and metacognitive strategies, but not social-behavior strategy, to be significant mediators between motivational regulation and achievement. While not rejecting those authors’ explanation – that their result was linked to their test-intensive research setting – we believe that social-behavior strategies may function as a moderator rather than a mediator. Specifically, we argue that students’ self-regulated social-behavioral strategy has two sides: one being a placebo that flattens the negative impact of performance-oriented motivational regulation on anxiety; and the other, a booster that accelerates that relationship. Karabenick (2004) found that students with performance-approach orientations paid close attention to the negative impacts or costs of seeking help, and thus avoided doing so, which in turn led them to have higher anxiety levels (Karabenick, 2004). This may help to explain our finding that, in the case of two students who reported the same high levels of performance self-talk, the one who relied more heavily on social-behavior strategy tended be less anxious.

Our finding that motivational-regulation strategies stably predicted both current and subsequent academic well-being was consistent both with prior literature (Dickhäuser et al., 2016) and our initial hypothesis that the predictive pattern of motivational regulation would be relatively stable. On the other hand, Social-behavior strategy only moderated the relation between performance self-talk and T1 anxiety, not subsequent anxiety. This indicates that the distal effect of social-behavior strategies on one’s well-being is rather limited, which is unsurprising insofar as such strategies are inherently short-term ones, i.e., aimed at tackling learning problems when and as they occur (Teng and Zhang, 2018).

Our study has several limitations that must be acknowledged. First, we collected students’ self-reported survey data as measurements of their motivational regulation, self-regulated writing strategies, and writing emotions. Further studies should consider triangulating students’ questionnaire responses *via* a range of other data-collection methods, including behavioral observation (Schwinger et al., 2009), interviews, journals, and/or thinking aloud (Greene et al., 2011). Second, while longitudinal data collection allowed us to visit and revisit the associations between key variables, the small (2-month) span of time between our two observation time-points could have limited our understanding of the effect of motivational regulation and self-regulated writing strategies on long term writing emotions. Future studies could therefore usefully extend the time spans of data collection. Third, causal inferences cannot be reached due to the lack of bi-directional reciprocal examination of the associations between variables. Fourth, the motivational regulation we captured was mainly performance-approach oriented rather than performance-avoidance oriented. Given the difference between these two orientations (Elliot, 1999; Pintrich, 2000), and previous findings about the maladaptive outcomes that a performance-avoidance orientation might be linked to Schwinger and Stiensmeier-Pelster (2012), future studies should consider differentiating between these two orientations when testing strategies’ impacts on writing emotions. Fifth, although our participants’ academic performance varied considerably, the whole sample was drawn from a prestigious learning institution at which most students are expected to possess relatively high levels of both motivation and learning ability. Future research should therefore test whether the associations found in this study can be replicated in fundamentally different learning contexts, especially ones where the students find L2 writing quite challenging. Last but not least, given that the purpose of this study was to identify predictors for L2 writing achievement emotions and their patterns of predictive power across two time points, we did not examine in more detail the internal constructs of motivation regulation and self-regulated strategy use, future research can validate and extend our findings by adopting more complicated analytical methods, such as structural equation modeling (SEM) and corresponding moderation analysis (e.g., Hayes’s PROCESS), to draw a more holistic picture of the relationship among motivation regulation, self-regulated strategy use, and L2 achievement emotions.

## Conclusion

Motivational regulation is generally considered an integral part of the SRL framework; and previous research has focused on types of motivational regulation, as well as its mediated relations with learning achievement. The present study looked beyond both of these perspectives, by reconsidering motivational-regulation strategies’ relations to both proximal and distal achievement emotions, as well as how SRL strategies interact with them. Its four key findings are as follows. First, all types of motivational-regulation strategy directly predicted positive emotion under a “the more the happier” principle. Second, as measured by lower levels of negative emotion, mastery-, emotional-, and interest-oriented motivational-regulation strategies appeared to work better than performance-oriented ones. Third, higher use of social-behavior strategy reduced the strength of the positive relationship between performance self-talk and anxiety. And last but not least, the power of motivational regulation to predict emotion at different time points was relatively stable.

Taken together, these findings illuminate the predictive relations among motivation regulation, self-regulated strategy use, and achievement emotions in L2 writing, making the present study another addition to the limited but pedagogically important research into the longitudinal investigation of L2 emotions in general (Dewaele, 2021b). Given the differentiating predictive effects of motivational self-talk and the interactive effects of motivation regulation and self-regulation strategies on L2 achievement emotions, it will be an intriguing avenue for future research to devise interventions targeting students’ motivational, metacognitive, and cognitive self-regulated strategies. Experimental designs will be particularly helpful in revealing whether the training of such strategies as mastery self-talk, interest enhancement, and emotion enhancement truly promote students’ emotional experience and well-being in the long run. We also encourage experimental endeavors on developing students’ social-behavior strategies as our findings indicate their observed effectiveness in counteracting the potential negative effects of high levels of performance self-talk.

## Data availability statement

The raw data supporting the conclusions of this article will be made available by the authors, without undue reservation.

## Ethics statement

The studies involving human participants were reviewed and approved by Tsinghua University. The patients/participants provided their written informed consent to participate in this study.

## Author contributions

YZ: literature review, draft, data collection, and analysis. LD: literature review and draft. Both authors contributed to the article and approved the submitted version.
