# Supplementary material for: A study of the impacts of motivational regulation and self-regulated second-language writing strategies on college students’ proximal and distal writing enjoyment and anxiety
Source: Front Psychol. 2022 Aug 9;13:938346. doi: 10.3389/fpsyg.2022.938346 (PMC9396036; doi:10.3389/fpsyg.2022.938346)
Supplement: Supplementary file 1 [file Data_Sheet_1.docx]

**Appendix I**

**Questionnaire on Students’ English Writing**

**Motivational regulation strategies**

***Mastery self-talk***

1. I persuade myself to work hard in writing courses to improve my writing skills and knowledge.
2. I persuade myself to keep on learning in writing courses to find out how much I can learn.
3. I persuade myself to work hard in writing courses to learn as much as possible.

***Performance self-talk***

1. I tell myself that I need to do better than others in writing courses.
2. I tell myself that it is important to practice writing in English to outperform other peers.
3. I tell myself that it is important to get good grades in writing courses.
4. I tell myself that it is important to practice writing to get good grades in writing courses.

***Interest enhancement***

1. I look for ways to bring more fun to the learning of writing.
2. I choose interesting topics to practice writing in English.
3. I connect the writing task with my real life to intrigue me.
4. I try to connect the writing task with my personal interest.

***Emotional control***

1. I tell myself not to worry when answering questions in writing courses.
2. I tell myself to keep on completing a writing task when I want to give it up in writing courses.
3. I find ways to regulate my mood when I want to give up a writing task in writing courses.

**L2 self-regulated writing strategies**

***Text processing***

1. When revising, I check grammar mistakes.
2. When revising, I check spelling and punctuation.
3. When revising, I check the structure for logical coherence.
4. When revising, I check the cohesiveness or connection among sentences.
5. When revising, I check whether the topic and the content have been clearly expressed.

***Idea planning***

1. I read related articles to help me plan.
2. I use the internet to search for related information to help me plan.
3. I think about the core elements of a good composition to help me plan.

***Goal-oriented monitoring***

1. When I learn English writing, I set up goals for myself in order to direct my learning activities.
2. I check my English learning progress to make sure I achieve my goal.
3. I evaluate my mastery of the content in writing courses.
4. I monitor my learning process in writing courses.
5. When I am writing, I tell myself to stick to my plan.
6. I set up a learning goal to improve my writing.

***Peer learning***

1. In writing courses, I brainstorm with peers to help me write.
2. I discuss with my peers or teachers to have more ideas to write.
3. I work with other students in writing courses.

***Feedback handling***

1. I am open to peers’ feedback on my writing.
2. I am open to teachers’ feedback on my writing.
3. I try to improve my English writing based on peers’ feedback.
4. I try to improve my English writing based on teachers’ feedback.

**Writing achievement emotions**

***Anxiety***

1. I get nervous while writing English.
2. I worry whether I can learn to write good English.
3. I get so nervous that I don’t even want to begin to write in English.

***Enjoyment***

1. I look forward to writing in English.
2. I am so happy about the progress I made in English writing.
3. I enjoy the challenge of writing in English.
4. I learn English writing more than required because I enjoy it.
